# The Environment and Cyanophage Diversity: Insights From Environmental Sequencing of DNA Polymerase

**DOI:** 10.3389/fmicb.2019.00167

**Published:** 2019-02-08

**Authors:** Jan F. Finke, Curtis A. Suttle

**Affiliations:** ^1^Department of Earth, Ocean and Atmospheric Sciences, The University of British Columbia, Vancouver, BC, Canada; ^2^Department of Microbiology and Immunology, The University of British Columbia, Vancouver, BC, Canada; ^3^Department of Botany, The University of British Columbia, Vancouver, BC, Canada; ^4^Institute for the Oceans and Fisheries, The University of British Columbia, Vancouver, BC, Canada

**Keywords:** cyanomyovirus, DNA polymerase, gp43, selection, ecotypes, gene content

## Abstract

Globally distributed and abundant cyanophages in the family Myoviridae have dsDNA genomes with variable gene content, including host-derived auxiliary metabolic genes (AMGs) that potentially can facilitate viral replication. However, it is not well understood how this variation in gene content interacts with environmental variables to shape cyanomyovirus communities. This project correlated the genetic repertoire of cyanomyoviruses with their phyologeny, and investigated cyanomyovirus ecotype distribution as a function of environmental conditions across locations and seasons. Reference cyanomyovirus genomes were compared for their overlap in gene content to infer phyologenetic distances, and these distances were compared to distances calculated based on DNA polymerase (gp43) gene sequences. In turn, gp43 partial gene sequences amplified from natural cyanophage communities were used to describe cyanomyovirus community composition and to assess the relationship between environmental variables. The results showed the following: (1) DNA polymerase gene phylogeny generally correlated with the similarity in gene content among reference cyanomyoviruses, and thus can be used to describe environmental cyanomyovirus communities; (2) spatial and seasonal patterns in cyanomyovirus communities were related to environmental variables; (3) salinity and temperature, combined with nutrient concentration were predictors of cyanomyovirus richness, diversity and community composition. This study shows that environmental variables shape viral communities by drawing on a diverse seed bank of viral genotypes. From these results it is evident that that viral ecotypes with their corresponding genetic repertoires underlie selection pressures. However, the mechanisms involved in selecting for specific viral genotypes remain to be fully understood.

## Introduction

Cyanobacteria are globally distributed and abundant pico-phytoplankton that are estimated to account for around 25% of the world’s primary production (William, 1994; Liu et al., 1997; Field et al., 1998; Partensky et al., 1999b; Weigele et al., 2007). In the oceans, they are numerically dominated by members of the genera *Synechococcus* and *Prochlorococcus* (Liu et al., 1998), which are infected and lysed by viruses (cyanophages), a process that influences cyanobacterial and viral diversity, as well as carbon and nutrient cycling (Wilhelm and Suttle, 1999; Weinbauer and Rassoulzadegan, 2004; Muhling et al., 2005; DeLong et al., 2006; Weitz and Wilhelm, 2012).

Cyanophages infecting members of *Synechococcus* spp. and *Prochlorococcus* spp. have circular, dsDNA genomes (Suttle, 2000) and appear to be primarily lytic (Clokie and Mann, 2006). All cyanophages isolated to date belong to the order *Caudovirales* and, depending on morphology, are assigned to one of the three families *Myoviridae, Podoviridae*, or *Siphoviridae* (Marston and Sallee, 2003; Weinbauer and Rassoulzadegan, 2004). Cyanophages within the family of T4-like *Myoviridae*, are referred to as cyanomyoviruses which are characterized by having long contractile tails and a comparably large host range that can span across genera (Suttle and Chan, 1993; Lu et al., 2001; Sullivan et al., 2003; Lindell et al., 2004). Their genome sizes range from 150 to >200 kb with variable gene content and many unknown genes. The variable gene content also includes several examples of host-derived auxiliary metabolic genes (AMGs) (Breitbart et al., 2007; Clokie et al., 2010) encoding proteins involved in photosynthesis, nutrient uptake and carbon metabolism (Mann et al., 2003; Sullivan et al., 2005; Dammeyer et al., 2008; Williamson et al., 2008). For AMGs studies have shown that they are expressed and can benefit viral replication (Bragg and Chisholm, 2008; Hellweger, 2009; Thompson et al., 2011). Recent studies on cyanophage isolates suggest that the gain and loss of AMGs represents an adaptation to selection pressures (Crummett et al., 2016) such as phosphate stress (Kelly et al., 2013). Consequently, the differences in gene content and the associated adaptations to niches diversifies cyanophages into ecotypes (Marston and Martiny, 2016).

Virus replication is a resource-intensive process with a higher relative demand for nitrogen and phosphorus compared to cellular organisms (Jover et al., 2014), and in some cases dependance on light (Kao et al., 2005; Clokie and Mann, 2006; Jia et al., 2010). As well, abiotic environmental factors can influence viral production, replication rate and degradation, including phosphate availability (Suttle and Chen, 1992; Wilson et al., 1996; Sullivan et al., 2005, 2010; Jia et al., 2010; Bachy et al., 2018) and light (Suttle and Chen, 1992; Noble and Fuhrman, 1997; Weinbauer et al., 1999). This is reflected in the data of Clerissi et al. (2014) that show a link between the distribution of prasinovirues and phosphate in the Mediterranean Sea. Considering the dependence of cyanophage replication on resource availability and the variability in gene content in cyanophage genomes, it suggests that differences in the repertoire of genes in cyanophages may shape the composition of cyanophage communities under challenging environmental conditions. Cyanophages have shown biogeographic patterns in marine environments (Wilson et al., 1999a; Marston et al., 2013; Chow and Suttle, 2015; Huang et al., 2015; Hanson et al., 2016; Marston and Martiny, 2016), but the relationship between the genetic repertoire and community composition of viruses in natural samples is challenging to study and remains largely unexplored.

Several genes have been used as markers to describe the composition of cyanomyovirus communities. One of these is *gp20*, which encodes the vertex portal protein in T4-like viruses, including many cyanomyoviruses. Along a longitudinal transect in the Atlantic Ocean, Wilson et al. (1999b) used denaturing gradient gel electrophoresis to show that in natural viral communities partial *gp20* sequences changed markedly, but showed less variation with depth. Similarly, community changes in *gp20* sequences occur across small distances and depths, as well as across seasons, and in association with changes in physical environments and cyanobacterial host communities (Frederickson et al., 2003; Wang and Chen, 2004; Muhling et al., 2005; Sandaa and Larsen, 2006). Despite pronounced seasonal and spatial variations in *gp20* sequences, some genotypes are surprisingly widely distributed across sharply different environments (Short and Suttle, 2005). However, McDaniel et al. (2006) found that *gp20* sequences could only be amplified from about 60% of cyanomyovirus isolates, while Short and Suttle (2005) suggested that the primers used to amplify *gp20* sequences were not specific for cyanophages, raising doubts about *gp20* being a suitable marker for cyanomyovirus diversity.

Another highly conserved gene (*gp23*) that encodes the major capsid protein has been used to examine diversity in myoviruses infecting a broad range of hosts from fresh and marine waters (Tetart et al., 2001; Filée et al., 2005; Comeau and Krisch, 2008; Butina et al., 2010). Studies showed resilience in taxonomic composition that covaried with bacteria if a two-day time lag was incorporated (Needham et al., 2013), and while some OTUs persisted there were strong seasonal patterns in the composition of viral communities (Chow and Fuhrman, 2012). A recent study also showed patterns of co-occurrence among phytoplankton, bacteria and myovirus communities (Needham et al., 2017); however, *gp23* is also conserved across other myoviruses, making it is less useful to specifically infer cyanophage diversity (Chow and Fuhrman, 2012).

Alternatively, the AMGs *psbA* and *phoH*, which encode core proteins involved in photosynthesis and phosphorus metabolism, respectively, have been used as marker genes for a broader range of viruses in freshwater and marine environments (Mann et al., 2003; Chénard and Suttle, 2008; Goldsmith et al., 2011, 2015b), either alone or in conjunction with other marker genes (Goldsmith et al., 2015b). However, both genes are host-derived and neither essential nor exclusive to cyanomyoviruses, and thus difficult to use in studying this specific group of viruses.

The DNA polymerase gene, *gp43*, has relatively recently been used as a marker gene for myoviruses, and while the primers were not designed to be cyanophage-specific they amplify cyanomyoviruses well (Marston and Amrich, 2009). An extensive study of cyanophage isolates revealed great diversity, seasonality and geographic variability in *gp43* sequences (Marston et al., 2013). The data also highlighted stark differences in community composition among contrasting environments, and that seasonal composition varied more gradually. Marston and Martiny (2016) extended these observations and used *gp43* to describe temporal patterns in cyanomyovirus ecotypes.

In contrast to using marker genes, phylogenies based on the presence and absence of genes across genomes can be used to compare closely related viruses and are the best way to describe differences in their genetic repertoire (Yutin et al., 2014; Legendre et al., 2015; Finke et al., 2017). However, this approach requires full genome sequencing and annotation of isolates and thus cannot be used to study complex natural communities.

Ideally, it would be possible to infer the similarity in gene content among natural communities of viruses from amplicon sequences of a conserved marker gene. Here, we establish a correlation between the gene content of cyanomyoviruses and sequences of the DNA polymerase gene, *gp43*. This correlation is used to interpret the distribution of cyanomyovirus genotypes and their associated gene content in relation to environmental conditions across locations and seasons. The data reveal spatial and seasonal patterns in the viral communities that are related to differences in environmental variables. Salinity and temperature, describing the mixing status of the water column, and the concentrations and accessibility of nutrients were significant predictors of cyanomyovirus richness, diversity and community composition. Understanding the relationship between environmental variables and viral community composition can help to predict the response and ecological impact of cyanomyoviruses against a backdrop of environmental change.

## Materials and Methods

### Sampling

A total of 42 environmental samples were collected. Eighteen samples were taken in the Strait of Georgia and adjacent waters (SOG), 24 were from Saanich Inlet (SAA) over a 12-month period from the mixed surface layer (2–5 m) and at 10 m depth. The 18 SOG samples covered a range of environments from 13 locations (Supplementary Figures 1A,B); seven were from open straits (Johnstone Strait, Queen Charlotte Sound, Port Elizabeth, Discovery Passage and Campbell River), and five were from sheltered inlets. For each SOG sample, six discrete water samples were collected from the surface throughout the mixed layer to the subsurface chlorophyll maximum, and combined, to provide an integrated sample representing the mixed layer; the bottom sampling depth varied from eight to 18 m (Supplementary Table 1). The 24 samples from SAA represent a seasonal cycle in the surface layer and at ten meters, over 12 months (Supplementary Figures 2A,B). The mixed layer depth in SAA ranged from two to below 10 m, with deeper mixing usually occurring in the fall and spring. Additionally, SAA is seasonally stratified, and partially separated from adjacent waters outside the inlet by an 80 m deep sill at its entrance, creating a low-oxygen deep layer. Twenty to 200 liters of water were sampled with GO-FLO^®^bottles (General Oceanics, Miami, FL) and processed immediately or stored at 4°C in the dark until processing within 24 h. Samples were pre-filtered through 2.7 μm nominal pore-size GF/D glass-fiber filters (Whatman GE Health Care, Little Chalfont, United Kingdom) and 0.22 μm pore-size Sterivex filters (Merck Millipore, Billerica, MA) for SAA samples and 47 mm diameter 0.7 μm pore-size GC50 glass-fiber and 0.45 μm pore-size HVLP filters (Merck Millipore, Billerica, MA) for the SOG samples. The remaining virus-size particulate matter in the samples was then concentrated to 500 ml by tangential flow ultrafiltration (TFF, Prep-Scale) using a 30 kDa-cutoff membrane (Merck Millipore, Billerica, MA). Viral concentrates (VCs) were stored at 4°C until used.

### Environmental Data Collection and Processing

Depth profiles of temperature and salinity were measured with a rosette-mounted or cable-deployed CTD SBE 25 (Seabird Electronics Inc., Bellevue, WA, United States). Salinity was measured by conductivity and expressed in PSU (Practical Salinity Units), temperature was measured in degree Celsius, and depth was measured by pressure and expressed in meters. Chlorophyll-a fluorescence was measured using a WETStar fluorometer (Seabird Electronics, Bellevue, WA, United States), and oxygen concentration was measured by a SBE 43 oxygen sensor (Seabird Electronics, Bellevue, WA, United States). Photosynthetically active radiation (PAR) was measured with a QSP-200PD sensor (Biospherical Instruments, San Diego, CA, United States).

On board, nutrient samples were filtered through 0.22 μm pore-size PVDF syringe filters and the filtrate stored at -20°C for later analysis with a Bran & Luebbe AutoAnalyzer 3 (SPX-Flow, Norderstedt, Germany) using air-segmented continuous-flow analysis. Combined nitrate (reduced to nitrite) and nitrite, phosphate and silicate were measured by absorbance following established protocols (Murphey and Riley, 1962; Armstrong et al., 1967).

Viral and bacterial abundances were measured using a Beckton Dickinson FACSCalibur flow cytometer with a 15 mW 488 nm air-cooled argon ion laser following the general protocols of Brussaard (2004) and Brussaard et al. (2010) for viruses, and Marie et al. (1999) for bacteria. Briefly, samples were fixed for 15 min at 4°C in the dark with 25% electron-microscopy grade glutaraldehyde (final concentration 0.5%), followed by snap-freezing in liquid nitrogen and storage at -80°C. Prior to measurement, samples were thawed and diluted in 0.2 μm filtered, autoclaved TE 10:1 buffer (10 mM-Tris HCl; 1 mM EDTA pH 8.0) and stained with SYBR Green I (Invitrogen, Carlsbad, CA) at a final dilution of 0.5 × 10^-4^ of the commercial stock, incubated for 10 min at 80°C or 15 min at room temperature for viruses and bacteria, respectively. Samples were diluted in TE buffer (pH 8.0) to ascertain 100 to 1000 events s^-1^ during measurements. Viruses and bacteria were discriminated by plotting green fluorescence against side scatter (SSC) and data were analyzed with CYTOWIN version 4.31 (Vaulot, 1989) and WEASEL version 3.3 (Battye, 2015).

### DNA Extraction, PCR and Sequencing Library Preparation

For DNA extraction, 25 ml of VC were syringe filtered through 0.22 μm pore-size GV PVDF Millex filters (Merck Millipore, Billerica, MA, United States) and centrifuged for 6 h at 120,000 *g* and 8°C. The supernatant was discarded and viral pellets were resuspended in 500 μl TE buffer at 4°C overnight. Free DNA was treated with 5 μl DNase I (Invitrogen, Carlsbad, CA, United States) at 37°C for 15 min and inactivated with 10 μl EDTA (0.25 M) at 65°C for 15 min. Viral capsids were lysed with 60 μl Proteinase K (Invitrogen, Carlsbad, CA, United States) at 56°C for 15 min; viral DNA was extracted with Pure Link Viral RNA/DNA columns (Invitrogen, Carlsbad, CA, United States) following the manufacturer’s instructions and eluted in UltraPure water (Invitrogen, Carlsbad, CA, United States).

To ensure an equal amount of template in the PCR, DNA was first quantified with a Qubit 2.0 using the dsDNA HS Assay Kit (Invitrogen, Carlsbad, CA, United States). Viral DNA polymerase gene (*gp43*) fragments of about 475 bp length were amplified by PCR using primers from Marston et al. (2013). For each sample, 1–2 ng of template DNA were used in a two-step, large volume PCR. PCR conditions were an initial denaturation step at 94°C for 3 min, a total of 35 cycles of denaturation at 94°C for 45 s, annealing at 50°C for 45 s, extension at 72°C for 45 s and a final extension at 72°C for 10 min. Triplicate PCR products were pooled and run on a 0.8% Ultrapure LMP Agarose gel (Invitrogen, Carlsbad, CA, United States). Bands in the appropriate size range of 475 bp were excised and the DNA extracted with the Zymoclean Gel DNA Recovery Kit (Zymo, Irvine, CA, United States) and eluted in UltraPure water (Invitrogen, Carlsbad, CA, United States). DNA products were quantified by Qubit, aliquoted and stored at -20°C.

For library preparation, 500 ng of DNA product was used with the NxSeq Low DNA AmpFREE kit (Lucigen, Middleton, WI, United States) following the manufacturer’s protocol with NextFlex-96 sequencing adapters (Bioo, Austin, TX, United States). Libraries were purified and size selected (∼600 bp) with Agencourt AMPureXP beads (Beckman Coulter, Pasadena, CA, United States) and eluted in low TE. Library construction was confirmed with a Bioanalyzer 2100 using High Sensitivity DNA Chips (Agilent, Santa Clara, CA, United States).

### Sequencing

For pooling, libraries were quantified by Q-PCR with SSoFast Eva Green Supermix (BioRad, Hercules, CA, United States) and KAPA DNA Standard (KAPA Biosystems, Boston, MA, United States) on a C10000 Touch PCR block with a CFX 96 head (BioRad, Hercules, CA, United States). Pooled for equal template concentration, libraries were sequenced in two rounds at the UCLA (Los Angeles, CA, United States) and McGill/Genome Quebec (Montreal, QC, United States) sequencing facilities using 2 × 300 MiSeq paired-end technology (Illumina, San Diego, CA, United States).

### Bioinformatic Processing

The reference phylogeny was built from the following 19 fully sequenced and annotated cyanomyovirus genomes (NCBI accession numbers): S-SM1 (NC_015282), S-SM2 (NC_015279), S-ShM2 (NC_015281), S-SSM7 (NC_015287), S-SSM5 (NC_015289), S-PM2 (NC_006820) S-RSM4 (NC_013085), Syn1 (NC_015288), Syn9 (NC_008296), Syn19 (NC_015286), Syn33 (NC_015285), P-HM1 (NC_015280), P-HM2 (NC_015284), P-SSM2 (NC_006883), P-SSM7 (NC_015290), P-SSM4 (NC_006884), P-RSM1 (NC_021071), P-RSM4 (NC_015283), P-TIM40 (NC_028663). To identify shared genes among viruses coding sequences (CDSs) were clustered with USEARCH 8.1 at a 50% amino-acid identity. Phylogenetic distance between each viral genome was calculated based on the presence or absence of shared CDSs with the formula Dij = -ln(Sij/sqrt(Ni*Nj)), with Sij, Ni, and Nj as the number of shared genes, number of genes in one genome and number of genes in the other genome, respectively (Yutin et al., 2014). A neighbor-joining (NJ) tree was built in the ape package (Paradis et al., 2004) in R (R Core Team, 2015). Inferred amino-acid sequences for full-length *gp43* were determined for each of the genomes above, aligned in Clustal (Sievers et al., 2011), and a maximum-likelihood (ML) tree built in RaxML 8.0.0 (Stamatakis, 2014) using the WAG substitution model, with the optimal substitution model selected in prottest-3.4 (Darriba et al., 2011). A Mantel Test to compare distance matrices based on gene presence/absence versus translated *gp43* sequences was performed in the ade4 package (Dray and Dufour, 2007) using R. Identities of partial gp43 amino-acid sequences from reference viruses were determined in RaxML 8.0.0., and their specificity to full-length sequences at different identity levels compared in R.

Sequences for environmental *gp43* amplicons were trimmed using TRIMMOMATIC 0.33 (Bolger et al., 2014), applying a quality thread score of 30 and a minimum length of 36 nt. Paired reads were merged with USEARCH 8.1 (Edgar, 2010), translated with FragGeneScan 1.20 (Rho et al., 2010), and size-selected for a minimum length of 140 amino acids. Reads from all samples were pooled and dereplicated, and then clustered and chimera tested with USEARCH 8.1 at 97% amino-acid identity based on the similarity of reference sequences. Singletons were removed and OTUs were selected for sequence similarity to cyanophages using BLAST-P with a cut-off E-value of 10^-3^. OTUs were placed on the full-length amino-acid gp43 ML reference tree by the Evolutionary Placement Algorithm (EPA) in RaXML 8.0.0. The phylogenetic tree was edited using iTOL v3.2.4 (Letunic and Bork, 2016), clades were defined by eye. Per sample, reads were parsed to representative OTUs with UPARSE 8.1 (Edgar, 2013) at an amino-acid identity of 97%.

### Statistical Analyses

Statistical analyses were performed in R. Scaling and principal component analysis (PCA) analysis of the environmental data was done using the FactoMineR package (Le et al., 2008). Parsed reads per sample were normalized to equal numbers of reads per sample; the VEGAN package (Oksanen et al., 2016) was used to determine diversity indices, and conduct the principal coordinate analysis (PCoA), analysis of similarities (ANOSIM), and canonical correspondence analysis (CCA). Environmental classification and subsequent indicator-species analysis were done in the IndecSpecies package (De Caceres and Legendre, 2009).

## Results

### Overview of Sequencing Results

**FIGURE 1:**
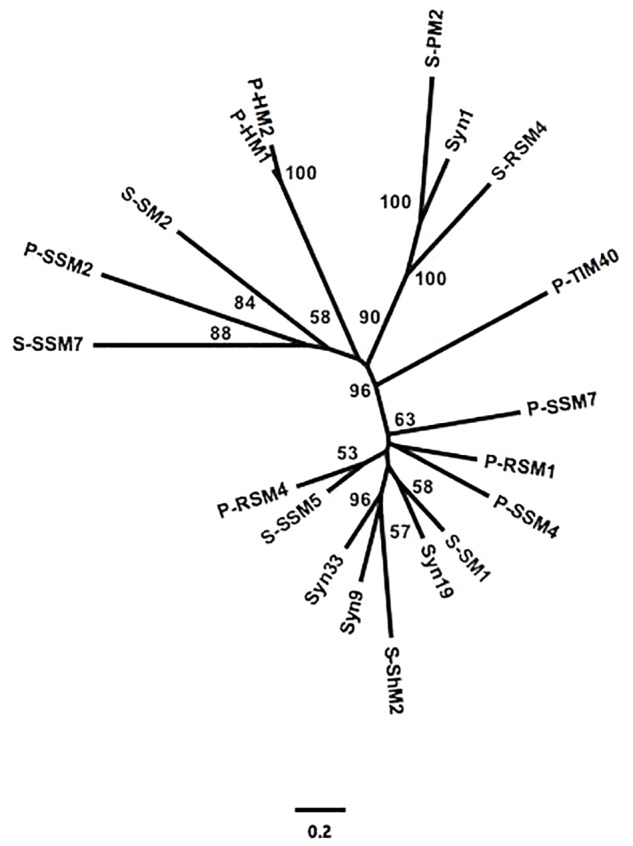
Neighbor-joining (NJ) phylogeny of reference cynaomyoviruses based on gene presence or absence. CDS of all genomes were clustered at 50% aa identity. Bootstrap values over 50% are shown.

**FIGURE 2:**
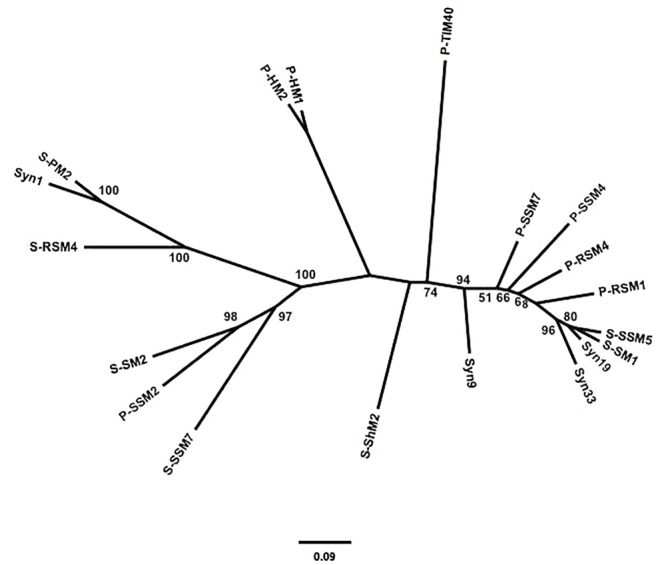
Maximum-likelihood (ML) phylogeny of reference cynaomyoviruses based on gp43 sequences. Phylogenetic distance is based in the full length gp43 amino-acid sequences. Bootstrap values above 50% are shown, scale is the substitution rate.

To assess the similarity between phylogenetic analyses based on DNA polymerase gp43 sequences and the gene content of T4-like cyanomyoviruses, the genomes of 19 reference cyanomyoviruses were compared based on their CDSs being clustered at 50% identity. A neighbor-joining (NJ) phylogenetic tree based on shared CDSs resolved several well-supported branches (Figure 1). In comparison to a maximum-likelihood (ML) tree based on corresponding full-length DNA polymerase amino-acid sequences (Figure 2) the trees displayed similar topography. A Mantel Test of the pairwise distances among reference viruses, between phylogenies based on gene content and DNA-polymerase sequences were significantly congruent (*r* = 0.87, *p* = 0.01) (Supplementary Table 2).

A comparison of the pairwise phylogenetic distances between the PCR-amplified region and full-length gp43 sequences from the 19 reference cyanomyoviruses demonstrated that variation between the phylogenetic distances calculated from full-length gp43 sequences approached zero when the amplicons above 95% amino-acid identity were clustered (Supplementary Figure 3). Furthermore, the highest observed pairwise identity among gp43 reference virus amplicons for these sequences was 96.4%. Accordingly, the environmental amplicons were clustered at 97% amino-acid identity.

**FIGURE 3:**
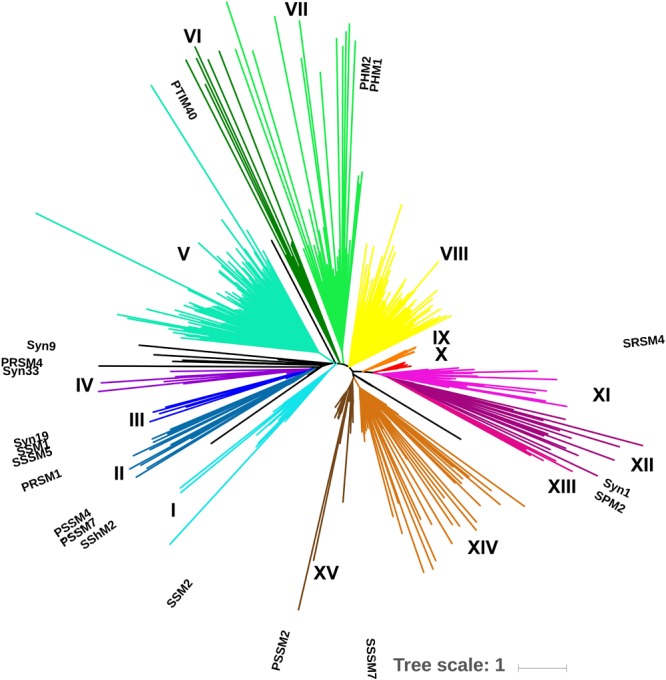
Environmental placement algorithm (EPA) phylogeny of 625 gp43 OTUs. A ML tree of full-length gp43 amino-acid sequences from 19 viruses served as a reference tree and 606 OTUs at 97% amino-acid identity from environmental amplicons were mapped onto the reference tree. Reference sequences are labeled; each tip represents an OTU. Clades are color coded and labeled in roman numerals; isolated OTUs are in black, the scale bar represents the substitution rate.

A total of 42 environmental samples were taken during 2010, 2011, and 2012. The combined sequencing data resulted in 9.84 million unique reads after quality control and dereplication; subsequent clustering at 97% amino-acid identity produced 12,200 gp43 OTUs. A minimum cluster size of 500 reads resulted in 667 OTUs, representing 97% of all initial, unique reads. BLAST-P analysis revealed that 606 of these OTUs (90.9%) were associated with cyanophages. An EPA tree constructed by placing the 606 OTUs on the maximum-likelihood reference tree of 19 full-length gp43 sequences revealed that most OTUs belonged to clades that were not represented by the reference sequences (Figure 3). Overall, the tree produced 15 distinct clades (I through XV), indicated by color.

### Overview of Environmental Conditions

**FIGURE 4:**
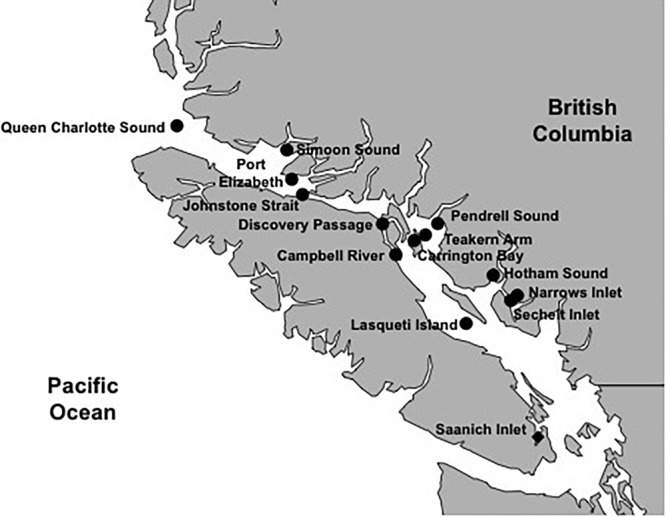
Sampling locations for areas within and adjacent to the Strait of Georgia (SOG) and Saanich Inlet (SAA). Samples were taken during 2010 to 2012 at several time points and depths in SAA (solid diamond) or integrated over depth for locations in and adjacent to the SOG (solid circles).

The 42 environmental samples, 18 in the SOG and 24 from SAA (Figure 4), covered a range of environmental conditions. The SOG samples ranged in salinity from 23 to 31 PSU; whereas in SAA the salinity remained around 28 PSU. Temperatures ranged from 9 to 15°C among SOG samples, while they varied seasonally in SAA between 7 and 14°C. Temperature (T) versus salinity (S) plots for SOG and SAA samples (Supplementary Figures 4, 5), show that density differences among samples in SOG were mainly driven by salinity, while in SAA it was a combination of salinity and temperature. The SAA samples from May 2011 and 2012 were characterized by higher chlorophyll and oxygen concentrations. For SAA, nutrient concentrations and viral and bacterial abundance data were only available for the 10 m samples. Picophytoplankton abundances measured by flow cytometry generally showed high abundances of *Synechococcus* spp., with some eukaryotes. To summarize, the environmental variables in the SOG samples showed that locations differed by salinity and nutrient concentrations; whereas, the SAA samples followed a seasonal pattern and were mainly defined by nutrient concentration and temperature (Supplementary Table 1).

### Variation Among Spatial Samples From the Strait of Georgia (SOG)

**FIGURE 5:**
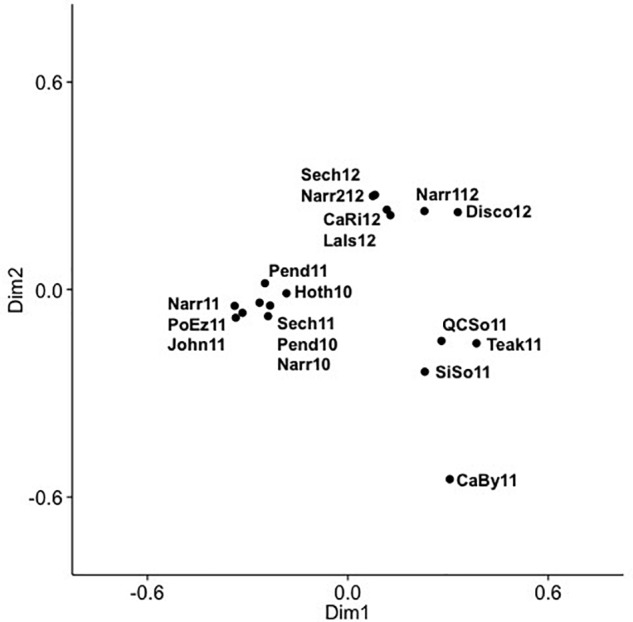
Principal coordinate analysis (PCoA) of cyanomyovirus community composition in SOG samples, labels show year and location: CaBy, Carrington Bay; CaRi, Campell River; Disco, Discovery Passage; Hoth, Hotham Sound; John, Johnston Strait; LaIs, Lasqueti Island; Narr, Narrows Inlet; Pend, Pendrell Sound; PoEz, Port Elizabeth; Sech, Sechelt Inlet; SiSo, Somoon Sound; QCSo, Queen Charlotte Sound.

Community composition of the combined 625 OTUs for the 18 SOG samples showed that viral communities had dominant and persistent OTUs within clades, as well as across phylogenetic groups (Supplementary Figure 6), and showed striking differences among the communities from Teakern Arm, Queen Charlotte Sound, Simoon Sound and Carrington Bay in 2011. Dominant OTUs for the SOG samples occurred in phylogenetic clades IV, V, VIII, XIV, XV. A PCoA of the SOG virus communities arranged samples into two main clusters and several outliers along two dimensions (Figure 5). Dimension one and two accounted for 29.9 and 19.7% of the community variation. The 2012 samples clustered together regardless of where they were collected. Another cluster comprised samples from 2010 and 2011, from inlet and strait locations. The four samples that fell outside of the two main clusters were from Teakern Arm and Carrington Bay in the center of the SOG sampling region and Queen Charlotte Sound and Simoon Sound to the north. As well, community composition in Narrows Inlet varied over the sampling years 2010, 2011, and 2012. Grouping samples by year, an ANOSIM analysis showed that 15% of the community variation could be explained by sampling year (*p* = 0.083). Samples from 2010 and 2012 showed low dissimilarity while dissimilarity was high for samples from 2011 and between years.

Diversity also varied across samples, with Port Elizabeth having the lowest estimated alpha diversity (2.27) and Queen Charlotte Sound the highest (4.98); beta diversity (Shannon) for the SOG samples was 1.96. Species richness was lowest in Narrows Inlet in 2010 (142) and highest in Discovery Passage in 2012 (494) (Supplementary Figures 9, 10 and Supplementary Table 3).

### Variation Among Temporal Samples From Saanich Inlet (SAA)

**FIGURE 6:**
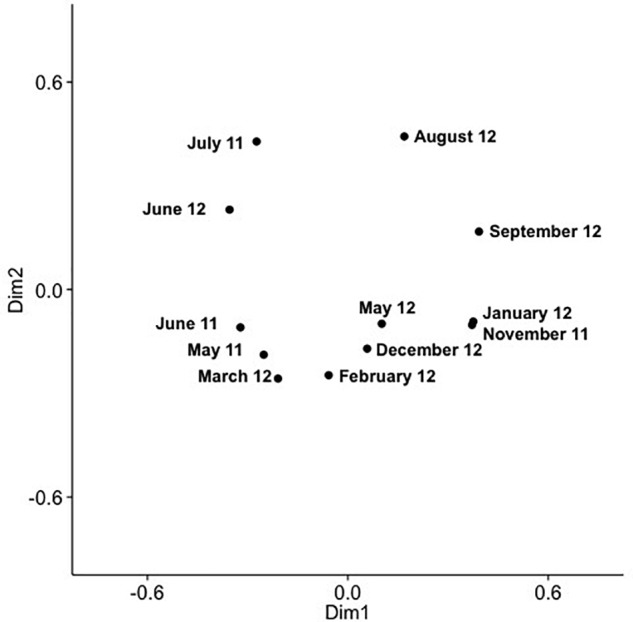
PCoA of SAA cyanomyovirus communities in samples from the surface layer; the PCoA is based on relative OTU abundances. Communities were rarefied; labels indicate sampling month and year.

**FIGURE 7:**
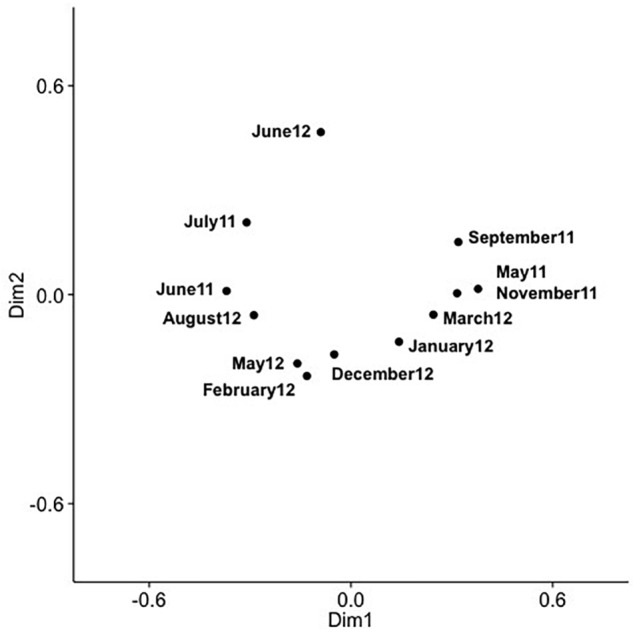
PCoA of SAA cyanomyovirus communities in samples from 10 m; the PCoA is based on relative OTU abundances. Communities were rarefied; labels indicate sampling month and year.

Analysis of community composition over 12 months for the surface and 10 m samples showed that some OTUs dominated throughout the year (Supplementary Figures 7, 8), and that these occurred in numerous phylogenetic clades, namely IV, V, VIII, X, XI, XIV, XV. Comparison of community composition across months for the surface layer and 10 m samples by PCoA in two dimensions revealed a seasonal pattern (Figure 6, 7), with dimensions one and two accounting for 31.5 and 24.5% of the community variation in the surface layer samples, and 39.4 and 21.3% for samples from ten meters. Communities from November to February clustered tightly together; whereas, communities from March through September were more spread out. Generally, the surface layer communities (Figure 6) showed more variation than communities from 10 m (Figure 7).

Diversity indices for the SAA surface layer samples ranged from 3.09 in August to 4.61 in February, while species richness ranged from 256 in January to 424 in March. At 10 m, the diversity ranged from 3.80 in May to 4.87 in December, and richness varied from 353 in March to 498 in December (Supplementary Figures 9, 10 and Supplementary Table 2). The beta diversity (Shannon) was 2.19 and 1.75 for the surface and 10 m samples, respectively.

### Combined Analysis in Relation to Environmental Variables

**FIGURE 8:**
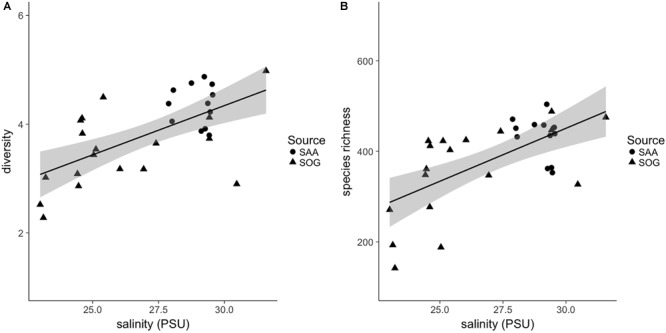
**(A)** Shannon alpha diversity and **(B)** species richness of combined samples in relation to salinity. Sample source is indicated as follows: SOG (solid triangles) and SAA 10 m (solid circles). For the linear regression, gray shading is the 95% confidence interval. For the diversity and richness correlations *R*^2^ and significance (*p*)-values are 0.38 and 0.39, respectively, and *p* = < 0.001 for both.

Combining the samples, the SOG samples had the largest range in diversity, followed by SAA surface layer and 10 m samples (Supplementary Figure 7). The beta diversity within sample subsets was highest in the surface layer samples from SAA (2.19), followed by SOG (1.96) and SAA at 10 m (1.75). The range in species richness was highest in the SOG samples, while on average the richness was highest in SAA 10 m samples (Supplementary Figure 8). A universal regression analysis of the combined alpha-diversity and species-richness data from the SOG and SAA 10 m samples, which had complementary environmental data, against the environmental variables showed a significant relationship to salinity (*R*^2^ = 0.38 and 0.39; *p* = < 0.001 and <0.001) (Figure 8).

**FIGURE 9:**
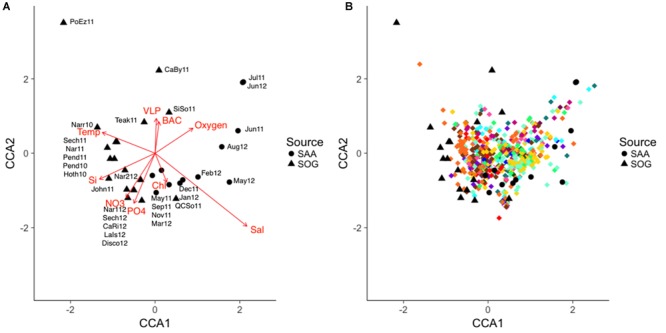
**(A,B)** CCA of the combined SOG and SAA 10 m cyanomyovirus communities. SAA samples are shown as solid circles and SOG samples as solid triangles, OTUs are shown as diamonds with colors corresponding to the phylogenetic clades in Figure 3. The direction and strength of the constraining environmental variables are shown in red. SOG labels: CaBy, Carrington Bay; CaRi, Campell River; Disco, Discovery Passage; Hoth, Hotham Sound; John, Johnston Strait; LaIs, Lasqueti Island; Narr, Narrows Inlet; Pend, Pendrell Sound; PoEz, Port Elizabeth; Sech, Sechelt Inlet; SiSo, Somoon Sound; Teak, Teakern Arm; QCSo, Queen Charlotte Sound. SAA labels denote sampling month and year.

Potential relationships between environmental variables and cyanophage community composition was explored by comparing similarity in community composition, covariation of OTUs and their relationship to environmental variables using the combined data from SOG and SAA (10 m) and a constrained correspondence analysis. Samples were scattered in two dimensions with dimension one (CCA1) describing 9.99% of the community variation and 27.09% of the accumulated variation, and dimension two (CCA2) describing 5.76% of the community variation and 15.61% of the accumulated variation (Figure 9). Communities were loosely spread out by sample location and season, with Port Elizabeth being distant to all other communities.

The combined environmental variables accounted for 36.9% of the constrained variation in a significant model (*p* = 0.04). Salinity was the strongest predictor, followed by temperature; both varied primarily along the first dimension, but in opposite directions. Nutrients concentrations co-varied primarily along the second dimension (Figure 9A). A stepwise regression of the model revealed temperature, salinity and nitrate as significant model parameters (*p* = 0.012, 0.018, and 0.047) influencing community composition. Figure 9B depicts the community composition in the CAA with the relative placement of the OTUs; OTUs are color coded to reflect their clade as depicted in Figure 4. Most OTUs clustered in the center, with some grouping with the SAA summer samples. OTUs from clades XIV and XV (brown colors), and VI and V (yellow and turquoise) clustered together while OTUs from other clades were spread out across the plot.

To further investigate whether specific OTUs defined communities, an indicator-species analysis was executed. Building on the results from the CCA, the combined samples were divided into three classes based on the strongest environmental variables, temperature, salinity and nitrate concentration. Environmental classes 1, 2 and 3 were predominantly characterized by high, medium and low mean nitrate concentrations, class 1 was relatively cold and saline while classes 2 and 3 were, on average, warmer and less saline. Each of the classes were about equally comprised of 10, 11 and 9 communities. Samples in the first class are the winter samples from SAA and well-mixed SOG samples. The second class comprised sheltered inlet samples from SOG, and the third class included the spring and summer samples from SAA, plus samples from Simoon Sound and Pendrell Island in 2010. An indicator species analysis on the three classes assigned 104 significant OTUs at a cut-off of *p* = 0.05, and revealed a number of indicator species with a significant association to one of the classes (Table 1). Most indicator species were assigned to the 3^rd^ class (80); only 6 and 18 were assigned to the 2nd and 1st classes, respectively.

**Table 1:** Cyanomyovirus indicator species analysis.

Class	1st	2nd	3rd
Temperature (°C)	9.1	12.9	11.2
Salinity (PSU)	29.1	24.8	28.1
Nitrate (μM)	25.1	11.8	4.7
Sample size	10	11	9
Assigned OTUs	18	6	80

*Environmental classes one, two, and three with their corresponding mean temperature, salinity, and nitrate concentration, and the class sample size and number of assigned OTUs per class that showed a significant association.*

## Discussion

This study examined differences in cyanophage community composition across a number of locations spanning a range of environmental conditions. Linking similarity in gene content of cyanophages to genetic distance as reflected by DNA polymerase (gp43) amplicon sequences allowed temporal and spatial changes in cyanophage community composition in the context of environmental conditions to be explored.

### Deriving Similarity in Gene Content From gp43 Sequences

Determining whether the genetic repertoire of cyanomyoviruses can be inferred from gp43 sequences first required a phylogenetic distance analysis based on the presence and absence of genes. This is a powerful approach to compare closely related viruses, as was done for viruses infecting eukaryotic phytoplankton (Yutin et al., 2014; Legendre et al., 2015). A neighbor-joining tree based on the CDSs from 19 reference cyanomyovirus genomes clearly separated viruses into distinct branches, and produced reasonable bootstrap support values that are comparable to those obtained for a similar analysis of viruses infecting eukaryotic phytoplankton (Yutin et al., 2014; Legendre et al., 2015; Figure 1). In comparison, the tree topology matched that of a maximum-likelihood-based phylogeny on full-length cyanomyovirus DNA polymerase (gp43) sequences (Figure 2), with the same viruses clustered together on branches or being similarly isolated on both trees. This congruence was further supported by the Mantel Test result of 0.87 between pairwise phylogenetic distances based on gene content and full-length gp43 sequences. The findings based on the 19 reference cyanomyovirus genomes suggest that similarity in gp43 sequences correlates to similarity in gene content of cyanomyoviruses.

Given that similarity in gene content among cyanomyoviruses can be inferred from gp43 sequences, the next step was to determine if gp43 amplicon sequences represent full-length gp43 sequences. The variance in pairwise distances of full-length gp43 sequences approached zero at an identity above 95% for amplicons, as well the highest observed identity of amplicon sequences was 96.4%. Consequently, amplicon sequences clustered at an amino-acid identity of 97% accurately reflected full-length sequences. This is relatively stringent compared to the 95% amino-acid identity that was used to cluster myovirus gp23 sequences (Chow and Fuhrman, 2012), but less stringent than the 99% nucleotide identity used for comparing gp43 sequences of myovirus isolates (Marston et al., 2013). We propose that this level of inferred amino-acid identity for gp43 amplicon sequences reflects full-length sequences without greatly inflating the diversity of environmental amplicons. With this approach, environmental gp43 amplicon sequences can be used as a proxy for similarity in gene content of cyanomyoviruses, and thus be used to create meaningful OTUs from environmental sequencing data.

### Environmental Variables Are Defining the Spatial and Temporal Samples

The 42 samples in this study spanned a range of conditions found in temperate coastal environments in which *Synechococcus* spp. and their phages occur. The variation in conditions among SOG samples was reflected by salinity differences, characteristic of coastal environments with significant freshwater inflow. In contrast, variation among SAA samples was primarily associated with temperature, in a seasonal pattern typical of temperate coastal waters, and with higher chlorophyll and oxygen concentrations in the May 2011 and 2012, associated with the spring bloom. The assessed environmental variables are all known to influence virus-host interactions (Mojica and Brussaard, 2014). Yet PAR, which showed very high daily variability was excluded from the analysis. Based on the flow cytometric signature, the cyanobacterial communities were dominated by *Synechococcus* spp., as found in similar environments (Partensky et al., 1999a; Scanlan and West, 2002). Overall, the observed link between nutrient availability, and salinity and temperature suggests that mixing of the water column is an important factor influencing the environmental conditions in these samples. This in turn affects viruses directly or through their hosts, as summarized in Mojica and Brussaard (2014).

### Phylogenetic Groups Across Samples

With a robust method of inferring similarity in gene content of cyanomyoviruses from gp43 amplicon sequence data, it was possible to study their diversity and genomic similarity in environmental samples. Clustering of environmental gp43 amplicon sequences from all samples at 97% amino-acid identity revealed 12,200 OTUs, consistent with earlier studies showing high phylogenetic diversity of myoviruses and cyanophages using gp20, gp23 and *phoH* amplicon sequences (Filée et al., 2005; Short and Suttle, 2005; Comeau and Krisch, 2008; Butina et al., 2010; Goldsmith et al., 2015b). Notably, 667 (5.5%) OTUs encompassed ∼97% of all sequences, showing extreme unevenness of myovirus genotypes, with an overwhelming preponderance of rare taxa. Further, a BLAST-P analysis identified cyanophages as the top hit for 606 (90.9%) of the OTUs, with the rest presumably being other T4-like phages that may or may not infect cyanobacteria. These results support the use of gp43 as a marker gene for examining cyanomyovirus diversity in environmental samples (Marston and Amrich, 2009).

However, the amplicons were not long enough to represent such a large diversity of sequences in a well-supported maximum-likelihood tree. Hence, adopting an EPA approach, where full-length gp43 sequences were used to construct a reference tree, onto which the 606 amplicon sequences were recruited, allowed the environmental sequences to be placed in a robust phylogenetic context (Figure 4). Given that gp43 sequences correlate to and reflect the similarity in gene content among cyanomyoviruses, the great richness depicted in the EPA tree also implies substantial variation in gene content in the environment. The clades associated with the 19 reference viruses are well represented by numerous environmental reads, but there are also many clades with no reference, indicating a great genomic richness of cyanomyoviruses that remains to be studied. Comparing community composition across locations and seasons for SOG and SAA samples shows some closely related OTUs were relatively abundant, widespread and persistent. Similarly, other studies have reported that some phage taxa are widely distributed and commonly found over a range of samples (Wilson et al., 1999a; Short and Suttle, 2005; Goldsmith et al., 2011; Chow and Suttle, 2015). Other recent studies on the dynamics of virus communities showed that some genotypes vary over time, while others consistently dominate (Chow and Fuhrman, 2012; Needham et al., 2013; Goldsmith et al., 2015b). The PCoA plots show large community differences in SOG, and more gradual shifts over seasons in SAA, which may reflect contrasting environmental conditions across locations versus more incremental seasonal transitions, similar to earlier observations on cyanophage communities (Chow and Fuhrman, 2012; Marston et al., 2013).

A more detailed look at the SOG data shows a pattern of alternating communities, with only one or the other community occurring and fine variations in OTUs within the dominant clades, similar to the fine scale diversification observed within clusters of cyanomyovirus isolates over time and space (Marston and Martiny, 2016). The comparison of SOG communities by PCoA, showed samples with similar community composition resolved into three main clusters (Figure 5), which were not well described by geographic proximity or the type of environment, nor did they match the pattern of environmental conditions. The similarity of the Queen Charlotte Sound and Simoon Sound communities can be explained by their proximity and both being relatively cold and well mixed. However, Teakern Arm and Carrington Bay were environmentally similar to each other but very different than Queen Charlotte Sound and Simoon Sound; yet also clustered in that group. That all samples from 2012 clustered together was also surprising, since they spanned a range of environments from sheltered Sechelt and Narrows Inlets to the more exposed SOG, perhaps reflecting larger annual weather patterns. As well, annual samples from Narrows Inlet over three years were substantially different in composition, indicating the communities are dynamic and environmentally influenced. The ANOSIM analysis did show that the variation in community composition among samples could partially be explained by sampling year. This could be explained by larger weather patterns leading up to the time sampling. Strong differences in community composition observed among some samples are consistent with earlier observations from B.C. coastal waters and geographically distant locations (Frederickson et al., 2003; Marston et al., 2013). While environmental conditions appear to be important in dictating cyanophage community composition from different locations and years in the SOG data, the observed patterns cannot be fully explained by the measured environmental variables.

Comparing changes in community composition of the SAA surface and 10 m samples by PCoA was consistent with community composition following a seasonal pattern (Figure 6, 7). Here, the community dynamics matched changes in environmental variables over seasons, in particular temperature, salinity and nutrient concentration. Changes in community composition in the surface samples were more pronounced than those from 10 m, presumably due to stronger exposure to seasonal fluctuations in the near-surface water. This matches findings from the Bermuda Atlantic Time-series Study, in which viral communities were less similar between the surface and 100 m under stratified summer conditions than under well-mixed winter conditions (Goldsmith et al., 2015a), with temperature and salinity, and to some degree chlorophyll, being significant predictors. The described seasonal pattern at SAA is also similar to observations of temporal myovirus (Chow and Fuhrman, 2012) and bacterioplankton (El-Swais et al., 2015) communities being most similar when sampled a year apart. The observed gradual temporal shifts with seasonal re-occurrence of community composition (Marston et al., 2013), and community compositions that vary with season and temperature (Wang and Chen, 2004) have also been observed in other studies of marine viruses. In contrast, very pronounced changes in community composition with season have also been reported (Sandaa and Larsen, 2006).

Overall, the dominance of different phylogenetic groups of phages could be explained by a difference in host communities. However, since broad host ranges have been documented for several cyanomyoviruses (Lu et al., 2001; McDaniel et al., 2006; Hanson et al., 2016) we suggest that dominance may at least partially reflect differing gene content among cyanomyoviruses that provides a competitive advantage under specific environmental conditions.

### Diversity Indices Correlate With Environmental Variables

Comparing all samples, diversities vary with the mixing regime of the sampling sites, being lowest in samples from the sheltered, stratified layer of Port Elizabeth (2.27) and SAA surface in August (3.09), and from 10 m in SAA in May 2011 (3.80). In contrast, the highest diversity is found in well-mixed SOG Queen Charlotte Sound (4.98), SAA surface samples in February (4.61), and from 10 m in December (4.87). Similarly, the beta-diversity analysis among subsets of samples showed that surface samples from SAA, which are more exposed to seasonal changes than the 10 m samples, have the highest beta-diversity describing overall community variation. This observation is further confirmed by significant correlations between alpha diversity and richness of cyanomyovirus communities, and salinity in the combined SOG and SAA 10 m samples (Figure 8). Notably, these correlations are significant despite combining samples from a range of environments. Similarly, Estrada et al. (2004) showed an increase in phytoplankton and picoplankton diversity over a salinity range from 15 to 25 PSU, while Herlemann et al. (2011) showed a slight increase in bacterial richness at salinities from 5 to 13 PSU. Salinity is a prominent factor in coastal water column stratification, thus the here presented data could mean that environments with higher salinity and a more mixed water column promote a higher diversity in cyanomyovirus communities, as it has been shown for phytoplankton communities in a lake, with high diversity being a transient state, occurring at times of relatively high mixing depths (Weithoff et al., 2001).

### Differences in Community Composition Show Relationships to Environmental Variables

To further identify key environmental variables associated with shifts in community composition, a canonical correspondence analysis (CCA) was performed on the combined SOG and 10m data from SAA (Figure 9A,B). Overall, 36% of the variation in community composition across all samples was explained by a combination of environmental variables. Regression analysis identified salinity, temperature, and nitrate as significant factors. Other studies have found relationships of various degrees between virus community composition and environmental variables. For example, an examination of viral community composition using metagenomics data showed that location, season and depth, and presumably the associated conditions, were strong predictors of viral community composition (Hurwitz et al., 2014), and a large-scale study that mainly focused on temperature found a weak correlation between environmental variables and cyanomyovirus community composition (Huang et al., 2015). More so, a study on prasinovirus distribution in the Mediterranean Sea found that phosphate was the strongest variable explaining viral community composition (Clerissi et al., 2014). Considering the relatively high requirements of viruses for nitrogen and phosphorus, and that viral replication uses a substantial amount of host nutrients (Jover et al., 2014), it is not surprising that viral community composition is tied to nutrient concentrations. In the SOG and SAA samples, nitrate and phosphate co-varied strongly; hence, in our model nitrate might simply have stronger statistical power than phosphate, and thus generally describe nutrient availability. Moreover, because temperature and salinity control stratification they can also affect nutrient availability, strengthening this argument. In the CCA most OTUs are clustered near the center, suggesting that communities vary by relative abundance of each OTU rather than by their presence or absence. This variation in relative abundance and not presence-absence has also been suggested by Rodrigues-Brito et al. (2010) and Needham et al. (2013).

Counterintuitively, the recorded biological variables (viral abundance, bacterial abundance and chlorophyll concentration) were not significant predictors of diversity or community composition. While studies have found correlations between bacterial abundance and viral abundance or viral lysis (Mojica et al., 2016; Wigington et al., 2016; Finke et al., 2017) results from studies examining cyanophage diversity and viral or cellular abundances have been inconsistent. One study along a north-south transect in the Atlantic Ocean found that cyanomyovirus diversity was not correlated to any environmental variable, but was correlated to host diversity (Jameson et al., 2011). Similarly, in the Red Sea cyanophage abundance and diversity covaried with the diversity and abundance of cyanobacteria (Muhling et al., 2005). Huang et al. (2015) found a correlation between cyanomyovirus communities and host communities based on reads recruited to isolates, but not with reads recruited to genotypes. Notably, for cyanopodoviruses, which appear to have a narrower host range than cyanomyoviruses, that relationship was strong in both cases (Huang et al., 2015).

We also undertook an indicator-species analysis to identify OTUs that are characteristic of specific environmental conditions (Table 1). Samples were divided into three environmental classes based on salinity, temperature and nitrate, the significant variables in the CCA analysis. The first class represents relatively cold and saline, well-mixed environments with high nutrient availability, the second and third classes represent environments with lower nutrient availability. The distribution of indicator species to the three environmental classes shows that while some phage OTUs were specific to well-mixed environments with high nutrient availability, more OTUs were specific to the low nutrient environment. These OTUs which were strongly correlated to the stratified environments suggest that some phages are specifically associated with lower nutrient environments, having traits that allow them to compensate for low-nutrient availability. However, at this stage we could not link such presumed traits to specific phylogenetic clades. Variable gene content in cyanomyoviruses is a mechanism that potentially allows some viruses to be selected for under specific, challenging environmental conditions. This is in line with the recent definition of ecotypes for viral isolates by Marston and Martiny (2016).

This study established that sequence differences in the DNA polymerase gene (*gp43*) of cyanomyoviruses correlates to differences in gene content among viruses; thus, gp43 sequences can be used to infer genetic diversity in cyanomyoviruses. Yet, only a fraction of the vast diversity was abundant in the cyanomyovirus communities across samples. Variance in cyanomyovirus community richness, diversity and composition was tied to salinity, temperature and nitrate concentration. While salinity can directly affect phage infectivity (Kukkaro and Bamford, 2009), here it likely reflects a complex combination of factors, including mixing and the associated availability of nutrients and exposure to sunlight. This in turn can select for viruses with complementary genes. A transect in the Atlantic Ocean found a significant correlation among temperature, salinity, nutrients and vertical mixing, all associated with a decreased lysis by phytoplankton viruses (Mojica et al., 2016). Moreover, based on an indicator species analysis, some viral OTUs were associated with specific environmental conditions, suggesting that their genetic repertoire gave them a competitive advantage, thus they represent viral ecotypes. There are many factors influencing viral communities including resident viral seed banks, and particle dispersal (Chow and Suttle, 2015), but interactions between cyanophages and their hosts are also likely influenced by environmental variables. These interactions are likely also influenced by the genetic repertoire encoded by viral ecotypes, which would allow specific virus genotypes to dominate under favorable environmental conditions.

## Data Availability Statement

The sequencing datasets for this study can be found in the NCBI-Short-Read-Archive (http://www.ncbi.nlm.nih.gov/bioproject/505679).

## Author Contributions

Both authors conceived and designed the project and conducted the field work. JF performed lab work, prepared sequencing libraries and oversaw sequencing, performed the analyses, and led the writing of the manuscript with input from CS.

## Conflict of Interest Statement

The authors declare that the research was conducted in the absence of any commercial or financial relationships that could be construed as a potential conflict of interest.
