# Supplementary material for: The Environment and Cyanophage Diversity: Insights From Environmental Sequencing of DNA Polymerase
Source: Front Microbiol. 2019 Feb 8;10:167. doi: 10.3389/fmicb.2019.00167 (PMC6375837; doi:10.3389/fmicb.2019.00167)
Supplement: Supplementary file 1 [file Data_Sheet_1.zip › Supplementary_Material_Proof/Supplementary_Material_Proof.docx]

Supplementary Material

Environmental variables shape cyanomyovirus communities

Jan F. Finke, Curtis A. Suttle*

*** Correspondence:** Corresponding Author: suttle@science.ubc.ca

# Supplementary Figures and Tables

## Supplementary Figures


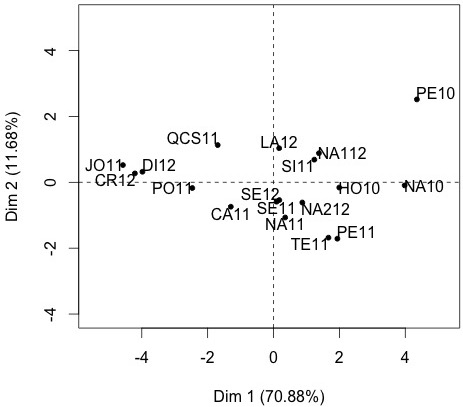


**Supplementary Figure 1a.** PCA (Principal Component Analysis) plot of the SOG (Strait of Georgia) samples. Labels: CaBy, Carrington Bay; CaRi, Campell River; Disco, Discovery Passage; Hoth, Hotham Sound; John, Johnston Strait; LaIs, Lasqueti Island; Narr, Narrows Inlet; Pend, Pendrell Sound; PoEz, Port Elizabeth; Sech, Sechelt Inlet; SiSo, Simoon Sound; QCSo, Queen Charlotte Sound; numbers indicate the sampling year.


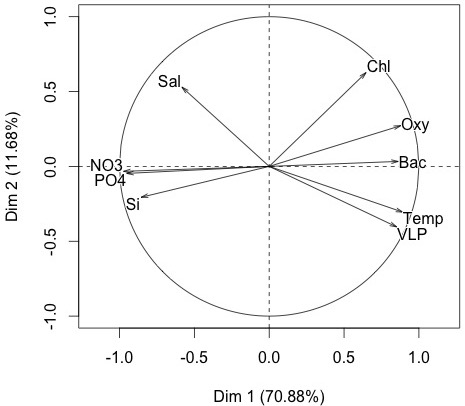


**Supplementary Figure 1b.** PCA (Principal Component Analysis) vector plot of the SOG (Strait of Georgia) samples.


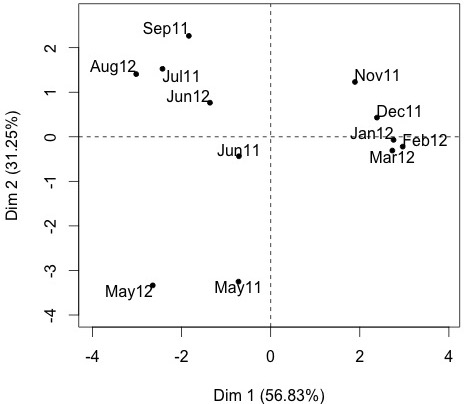


**Supplementary Figure 2a.** PCA (Principal Component Analysis) plot of the SAA (Saanich Inlet) 10 meters samples. Labels indicate the sampling month and year.


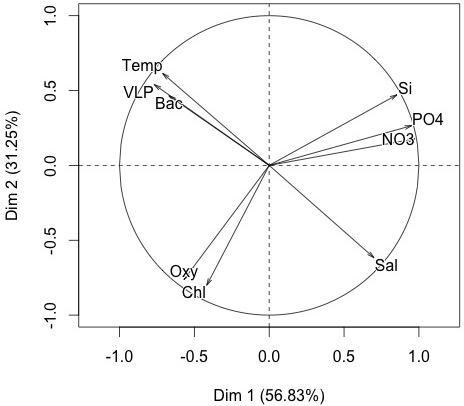


**Supplementary Figure 2b.** PCA (Principal Component Analysis) vector plot of the SAA (Saanich Inlet) 10 meters samples.


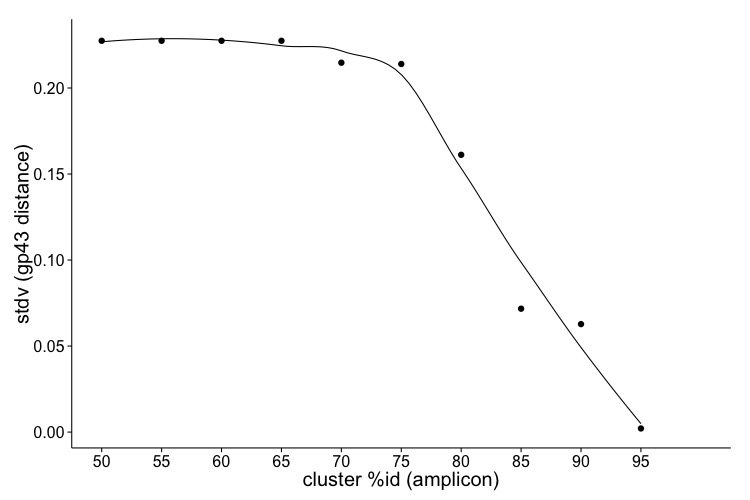


**Supplementary Figure 3.** Variation in pairwise phylogenetic distance of gp43. The variance is for full length gp43 sequences at different % clustering thresholds of the gp43 amplicons. Based on 19 reference cyanomyoviruses.


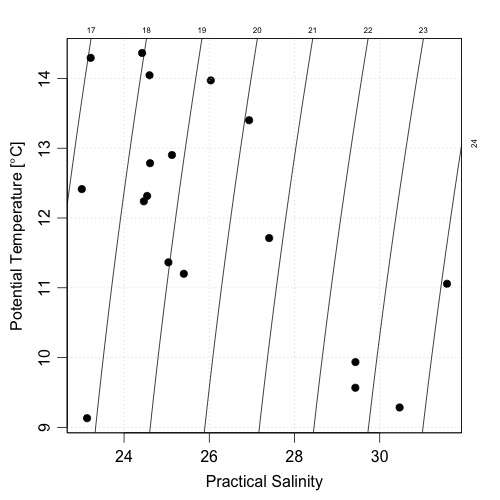


**Supplementary Figure 4.** Temperature-Salinity (TS) plot of the SOG (Strait of Georgia) samples, labels indicate sampling month and year.


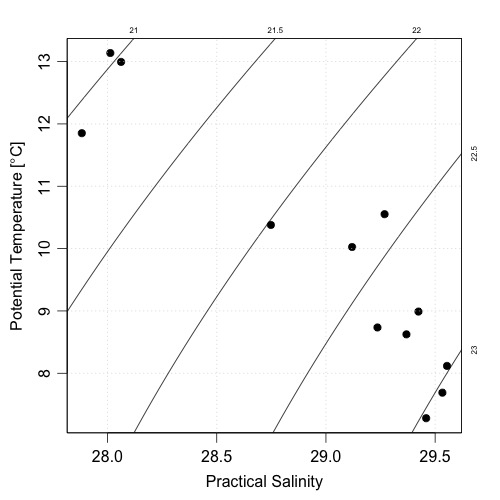


**Supplementary Figure 5.** Temperature-Salinity (TS) plot of the SAA (Saanich Inlet) 10 meters samples.


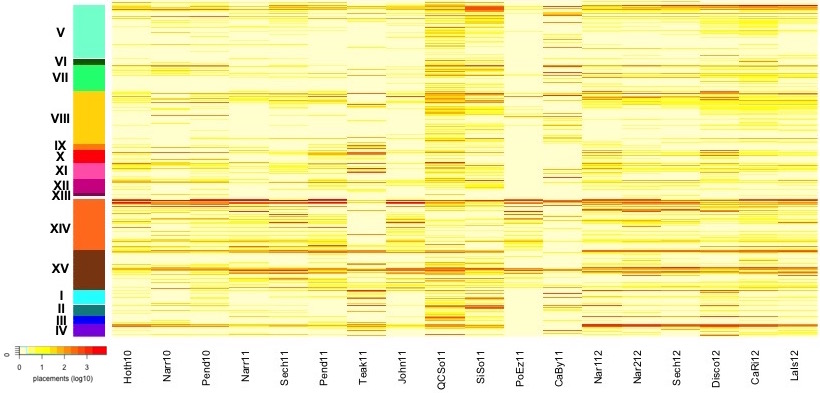


**Supplementary Figure 6.** Community composition of SOG (Strait of Georgia) samples. Communities were rarefied, heat indicates relative abundance. Samples are arranged in columns by year, OTUs are arranged in rows with clades indicated by color and number in correspondence to figure 4. Labels: CaBy, Carrington Bay; CaRi, Campell River; Disco, Discovery Passage; Hoth, Hotham Sound; John, Johnston Strait; LaIs, Lasqueti Island; Narr, Narrows Inlet; Pend, Pendrell Sound; PoEz, Port Elizabeth; Sech, Sechelt Inlet; SiSo, Simoon Sound; QCSo, Queen Charlotte Sound; numbers indicate the sampling year.


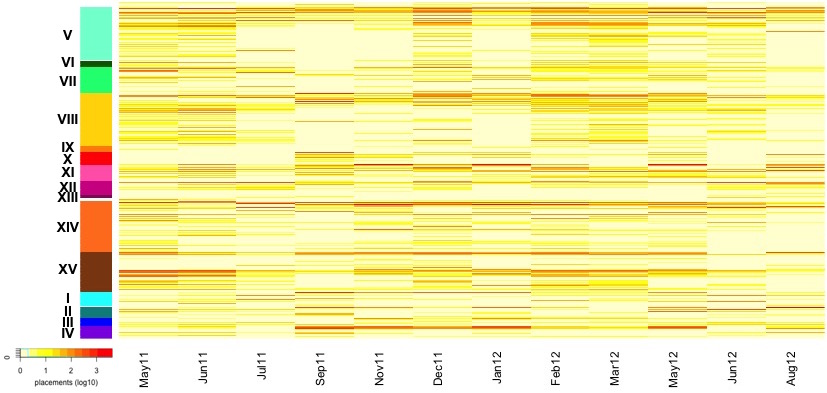


**Supplementary Figure 7.** Community composition of SAA (Saanich Inlet) samples. Samples from the surface layer, communities were rarefied, heat indicates relative abundance. Samples are arranged in columns by year, OTUs are arranged in rows with clades indicated by color and number in correspondence to figure 4, labels indicate sampling month and year.


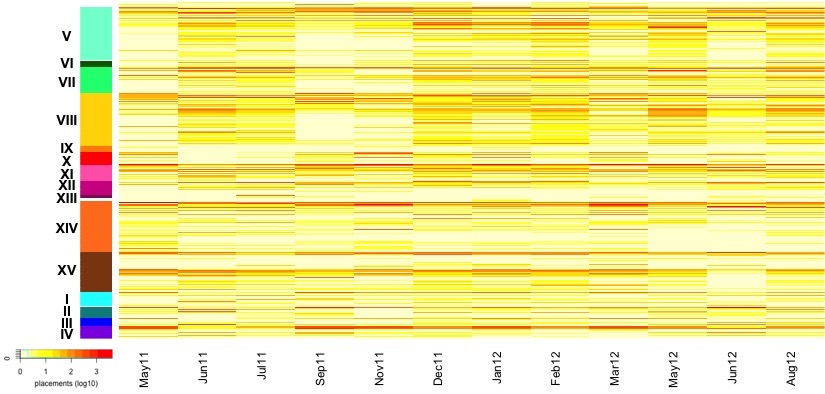


**Supplementary Figure 8.** Community composition of SAA (Saanich Inlet) samples. Samples from 10 m depth, communities were rarefied, heat indicates relative abundance. Samples are arranged in columns by year, OTUs are arranged in rows with clades indicated by color and number in correspondence to figure 5, labels indicate sampling month and year.


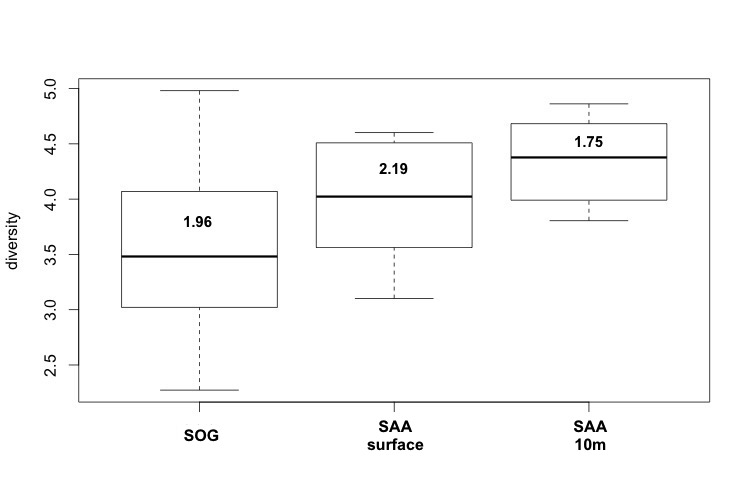


**Supplementary Figure 9.** Range of diversity for SOG (Strait of Georgia), SAA (Saanich Inlet) surface and 10 m communities. Diversity is Shannon alpha diversity, overall beta diversity per subset is shown in the box. Whiskers indicate the range, box 50 % of data points with median.


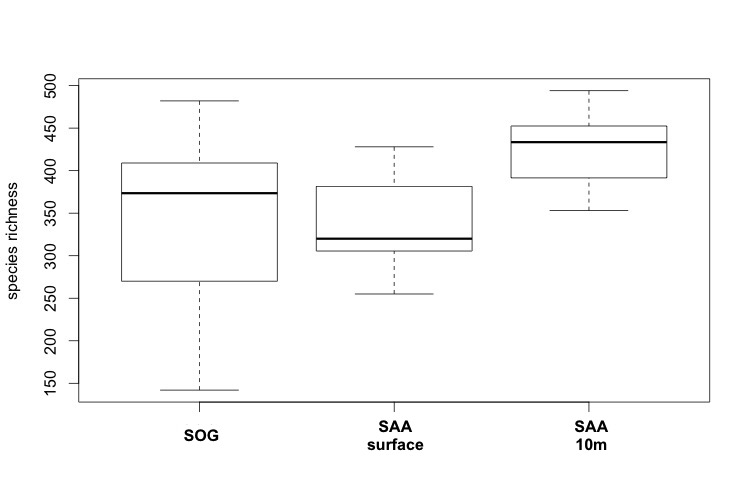


**Supplementary Figure 10.** Range of richness for SOG (Strait of Georgia), SAA (Saanich Inlet) surface and 10 m communities. Whiskers indicate the range, box 50 % of data points with median.

## Supplementary Tables (Captions)

**Supplementary Table 1.** Pairwise comparison of phylogenetic distances. Phylogenetic distance is based on gene content and full length gp43. Congruence was tested with a Mantel Test. Numbers of CDS per virus used in the cluster analysis are shown along the diagonal in italics.

**Supplementary Table 2.** Sampling details for gp43 environmental samples. Sampling locations and years are given, environmental parameters were measured *in situ* by CTD or analyzed in the lab.

**Supplementary Table 3.** Diversity indices of SOG (Strait of Georgia) and SAA (Saanich Inlet) samples. Diversity is Shannon alpha diversity, richness is defined as species richness. SOG samples arranged by site, SAA by month for surface and 10 m samples.
